# P02.98. Effect of selected plant extracts on haemozoin concentration in malaria patients

**DOI:** 10.1186/1472-6882-12-S1-P154

**Published:** 2012-06-12

**Authors:** U Dibua, A Kalu

**Affiliations:** 1 University of Nigeria, Nsukka, Enugu, Nigeria

## Purpose

To extract haemozoin from the blood sample containing malaria parasites and to screen for and compare both conventional antimalarial drugs and selected plant extracts.

## Methods

Haemozoin was extracted from blood samples of all the malaria positive patients studied by centrifugation, and the concentration was analyzed spectrophotometrically at 400nm wavelength. Comparative anti-malaria activity of some conventional antimalarials, including Maldox, Halfan, Artecxin, Amatem, Mefloquine (quinolines) and Malmed, as well as the leaf and stem back extracts of some local plants (Sarcocephalius latifolius and Alstonia boonei), was evaluated to establish the most effective agent for malaria therapy. Each was administered to patients in each malaria episode, and the absorbance of haemozoin produced determined at 400nm wavelength. Packed cell volume (PCV) was estimated to establish the proportion of red blood cells before and after haemozoin production, using a microhaematocrit reader.

## Results

All the chemical antimalarial drugs used effected reduction in haemozoin concentration. However, Mefloquine (quinolines) showed the highest activity with a significant difference of p=0.01. The plant extracts similarly exerted significant reduction in the hemozoin concentration. However, Alstonia boonei extract was the most effective in haemozoin reduction (p<0.01). Of all the therapeutants (chemical and plant extracts) tested, Alstonia boonei stem back extract most significantly reduced haemozoin production (p<0.01).

## Conclusion

The potential use of Alstonia boonei stem back extract as an effective antimalarial is evident from the study in consonance with its use in folkloric medicine.

